# Detection of IGF2BP3, HOXB7, and NEK2 mRNA Expression in Brush Cytology Specimens as a New Diagnostic Tool in Patients with Biliary Strictures

**DOI:** 10.1371/journal.pone.0042141

**Published:** 2012-08-07

**Authors:** Hans Dieter Nischalke, Volker Schmitz, Carolin Luda, Katharina Aldenhoff, Cordula Berger, Georg Feldmann, Tilman Sauerbruch, Ulrich Spengler, Jacob Nattermann

**Affiliations:** 1 Department of Internal Medicine 1, University of Bonn, Bonn, Germany; 2 Center of Integrated Oncology Cologne-Bonn, Department of Internal Medicine 3, University of Bonn, Bonn, Germany; Queensland Institute of Medical Research, Australia

## Abstract

**Introduction:**

It is a challenging task to distinguish between benign and malignant lesions in patients with biliary strictures. Here we analyze whether determination of target gene mRNA levels in intraductal brush cytology specimens may be used to improve the diagnosis of bile duct carcinoma.

**Materials and Methods:**

Brush cytology specimens from 119 patients with biliary strictures (malignant: n = 72; benign: n = 47) were analyzed in a retrospective cohort study. mRNA of IGF-II mRNA-binding protein 3 (IGF2BP3), homeobox B7 (HOXB7), Forkhead box M1 (FOXM1), kinesin family member 2C (KIF2C) and serine/threonine kinase NEK2 was determined by semi-quantitative RT-PCR using the ΔCt method.

**Results:**

IGF2BP3 (p<0.0001), HOXB7 (p<0.0001), and NEK2 (p<0.0001) mRNA expression levels were significantly increased in patients with cholangiocarcinoma or pancreatic cancer. Median ΔCt values differed by 3.5 cycles (IGF2BP3), 2.8 cycles (HOXB7) and 1.3 cycles (NEK2) corresponding to 11-fold, 7-fold and 2.5-fold increased mRNA levels in malignant versus benign samples. Sensitivity to detect biliary cancer was 76.4% for IGF2BP3 (80.9% specificity); 72.2% for HOXB7 (78.7% specificity) and 65.3% for NEK2 (72.3% specificity), whereas routine cytology reached only 43.1% sensitivity (85.4% specificity). Diagnostic precision was further improved, when all three molecular markers were assessed in combination (77.8% sensitivity, 87.2% specificity) and achieved 87.5% sensitivity and 87.2% specificity when molecular markers were combined with routine cytology.

**Conclusions:**

Our data suggest that measuring IGF2BP3, HOXB7 and NEK2 mRNA levels by RT-PCR in addition to cytology has the potential to improve detection of malignant biliary disorders from brush cytology specimens.

## Introduction

It is a challenging task for gastroenterologists to distinguish between benign and malignant lesions in patients with biliary strictures [1]. Although current diagnostic imaging methods such as transabdominal ultrasonography (US), computed tomography (CT), and magnetic resonance imaging (MRI) achieve a rather high sensitivity for detecting bile duct pathology, they cannot reliably differentiate between malignant and benign biliary strictures [2]. Therefore, intraductal procedures, such as endoscopic retrograde cholangiography (ERC) with brushings for routine cytology (RC) and intraductal forceps biopsy are often applied to identify patients with biliary malignancy [3], [4], [5]. Unfortunately, even if intraductal diagnostic procedures are combined (cholangioscopy, brush cytology, fine needle aspiration cytology and forceps biopsy), sensitivity to diagnose malignant biliary strictures reaches only 20–65% [3], [6], [7], [8], [9], [10]. Newer techniques, e.g. the Spyglass Spyscope system, can improve the diagnosis of biliary strictures but require additional equipment, and thus are not available in many hospitals [11], [12].

Low diagnostic yields of established intraductal diagnostic techniques is partly due to the growth and differentiation pattern of bile duct cancer, which frequently comprises tumors with a high content of fibrotic tissue, making morphological diagnosis difficult. To improve diagnostic precision, detection of tumor-associated molecular markers in routine cytology specimens by highly sensitive RT-PCR assays has emerged as a new diagnostic tool [13], [14]. In a previous study, we described that RT-PCR based semi-quantitative measurement of candidate gene mRNA from intraductal brush cytology specimens enables to detect cholangiocellular carcinoma with high diagnostic precision [15]. However, this study was limited by a small sample size, lack of patients with pancreatic cancer, and a selection of candidate genes that are over-expressed in cholangiocarcinoma cell lines, the in vivo expression of which, however, still remains unclear.

Recently, Obama et al. analyzed global gene-expression profiles of 25 intrahepatic cholangiocarcinomas using tumor cell populations purified by laser microbeam microdissection and a cDNA microarray technique [16]. This approach identified more than 50 genes which were frequently up-regulated in biliary cancer. Up-regulated expression was most conspicuous for FOXM1, IGF2BP3 [KOC], KIF2C, and HOXB7. Moreover, Kokuryo et al. recently identified NEK2 as an additional gene with high expression in cholangiocarcinomas [17]. Increased gene expression of HOXB7, KIF2C, NEK2, FOXM1 and IGF2BP3 has also been reported for several other types of cancer [18], [19], [20], [21], [22], [23], [24], [25], [26], [27], suggesting that these genes may be associated with tumor growth in general. Of note, up-regulation of these genes has also been found in pancreatic cancers [28], [29], [30], [31], [32], [33].

All these genes exert pivotal roles in the control of proliferation and differentiation.

The Forkhead Box M1 (FOXM1) transcription factor is a key cell cycle regulator involved both in the transition from G1 to S phase and the progression to mitosis. Altered FOXM1 signaling is associated with a variety of different tumors [34], [35]. IGF2BP3 is an oncofetal RNA binding protein which regulates insulin-like growth factor-II (IGF-II) transcripts and is involved in the post-transcriptional regulation of cell proliferation during embryogenesis [36]. KIF2C is a member of the kinesin-13 subfamily of kinesin-related proteins involved in chromosome segregation and correction of kinetochore–microtubule interactions during mitosis. KIF2C is over-expressed in breast and gastric cancer cells [37], [38]. HOXB7 is over-expressed both at the mRNA and protein level in human biliary cancer specimens but not detectable in normal biliary epithelium [39].

NEK2, a member of the serine/threonine kinase family, has been implicated in regulation of centrosome separation and spindle formation. Over-expression of NEK2 in various carcinoma cell lines suggests involvement of NEK2 in tumor development [17].

Because of their prominent role in the control of cell proliferation and differentiation, we considered these proteins as potential new diagnostic markers for biliary cancer. Thus, in the present study we established semi-quantitative RT-PCR assays for the detection of IGF2BP3, NEK2, KIF2C, FOXM1, and HOXB7 mRNA in biliary brush cytology specimens and evaluated their diagnostic precision as diagnostic markers for malignant bile duct strictures.

## Materials and Methods

### Ethics Statement

The reported studies were approved by the Institutional Review Board of the Bonn University Ethics Committee (008/04). Written informed consent was obtained from the patients prior to sample collection. Samples were coded and data stored anonymously.

### Patients

A total of 119 Caucasian patients with bile duct strictures (51 women, 68 men, median age 71.0 (23–94) years) were enrolled into this study. None of these patients had evidence for IgG4-associated autoimmune cholangitis or pancreatitis. Further patient characteristics are summarized in table 1.

**Table 1:** Clinical characteristics of patients.

	malignant strictures	benign strictures
**Diagnosis** **n (%)**	**52 (72) Cholangiocarcinoma**	13 (27.7) Cholelithiasis
	22 (42.3) Klatskin’s Tumor	13 (27.7) Inflammation
	21 (40.4) Carcinoma of thecommon bile duct	9 (19.1) Primary SclerosingCholangitis
	5 (9.6) Carcinoma of theintrahepatic bile ducts	8 (17.0) Pancreatitis
	4 (7.7) Papillary Carcinoma	3 (6.4) Surgical trauma
	**20 (28) Pancreatic Cancer**	1 (2.1) Primary BiliaryCirrhosis
	18 (90) Carcinoma of thepancreatic head	
	2 (10) Intrapancreatic ductalcarcinoma	
Gender (m/f)	49/23	19/28
Age#	71.0±11.7	61.3±17.1
Bilirubin#	5.88±7.64	1.62±2.1
GGT#	528.2±591.8	331.9±420.8
ALT#	133.2±184.1	66.3±68.8
AST#	104.9±100.1	64.9±55.1
AP#	380.5±251.5	244.0±223.5
LDH#	253.3±254.7	236.4±109.4

# Mean ± SD;

GGT: Gamma glutamyl transpeptidase, ALT: Alanine aminotransferase, AST: Asparagine aminotransferase, AP: Alkaline phosphatase, LDH: Lactate dehydrogenase.

A patient was considered to have a malignant stricture if there was (1) cytologic or histologic evidence of malignancy confirmed by tissue sampling by ERCP, percutaneous biopsy, surgical exploration, or autopsy or (2) a clinical course (over at least 12 months after enrollment) suggesting malignancy on the basis of new radiographic abnormalities such as metastases, infiltration of the mass into large blood vessels, lymphadenopathy with positive findings in positron emission tomography or (3) tumor-related death (death certificate diagnosis).

Patients were considered to have benign strictures if they did not have any of the aforementioned findings both during complete initial work-up and a follow up examination at 12 months or later to exclude disease progression.

### Samples

Brush specimens were obtained from biliary strictures using standard cytology brushes (Uno brush, MTW, Wesel, Germany) at routine ERCP. The brushes were passed across the lesion using 3–6 to-and-fro movements. Smears for cytological examination were prepared on site as part of routine diagnostic work-up; the remaining adherent cell material was immediately washed from the brush with 350 µL of RLT lysis buffer (RNeasy-Kit, Qiagen, Hilden, Germany) supplemented with 1% beta-mercapto-ethanol (Sigma-Aldrich, St. Louis, MO, USA) under vortexing, then samples were centrifuged and stored at –80°C until further use.

### Routine Cytology

Cytopathologists with particular expertise for RC independently reviewed the RC, and were blinded to the clinical records without knowledge of the other test results. RC specimens were interpreted as either positive for malignancy, suspicious for malignancy, atypical, negative for malignancy, or with inadequate cellularity for interpretation.

### RNA Extraction and Reverse Transcription

Total RNA was extracted using the RNeasy Mini Kit (Qiagen, Hilden, Germany) according to the manufacturer’s instructions. The extracted RNA was eluted in 40 µL of RNAse-free water and stored at –80°C. Elimination of genomic DNA and reverse transcription was carried out using the QuantiTect Reverse Transcription Kit (Qiagen, Hilden, Germany) according to the manufacturer’s standard protocol.

### Quantitative Real Time PCR

For each PCR run 1 µL of the obtained cDNA was used as template. PCR was carried out in a final volume of 10 µL on a LightCycler instrument using the LightCycler FastStart DNA-Master SYBR Green 1 kit (Roche Molecular Diagnostics, Mannheim, Germany). The reaction mix contained a 3.5 mM concentration of MgCl_2_. Primers, corresponding to nucleotide sequences of HOXB7 (sense: 5′-TGCGAAGCTCAGGAACTGAC-3′, antisense: 5′-TCATGCGCCGGTTCTG-3′) and the house-keeping gene glyceraldehyde-3-phosphate dehydrogenase (GAPDH) (sense: 5′-AGGGGGGAGCCAAAAGGG-3′, antisense: 5′-TGCCAGCCCCAGCGTCAAAG-3′) were purchased from TibMolbiol (Berlin, Germany) and used at a final concentration of 0.5 µM each. For analysis of IGF2BP3, KIF2C, FOXM1 and NEK2 mRNA expression commercially available QuantiTect Primer Assays (Qiagen, Hilden, Germany) were used at a final primer concentration of 0.5 µM each. For all PCRs an identical amplification protocol was used consisting of an initial denaturation step at 95°C for 10 min, followed by 40 cycles (denaturation at 95°C for 2 sec, annealing at 60°C for 5 sec, extension at 72°C for 15 sec) and fluorescence acquisition at 72°C.

PCR products were identified by melting curve analysis (95°C for 10 sec, 65°C for 15 sec and a slow ramp (0.2°C/sec) to 95°C with continuous fluorescence acquisition). The LightCycler software version 3.5 was used in all PCR experiments.

### Semiquantitative Analysis of Target Gene Expression

**Figure 1:**
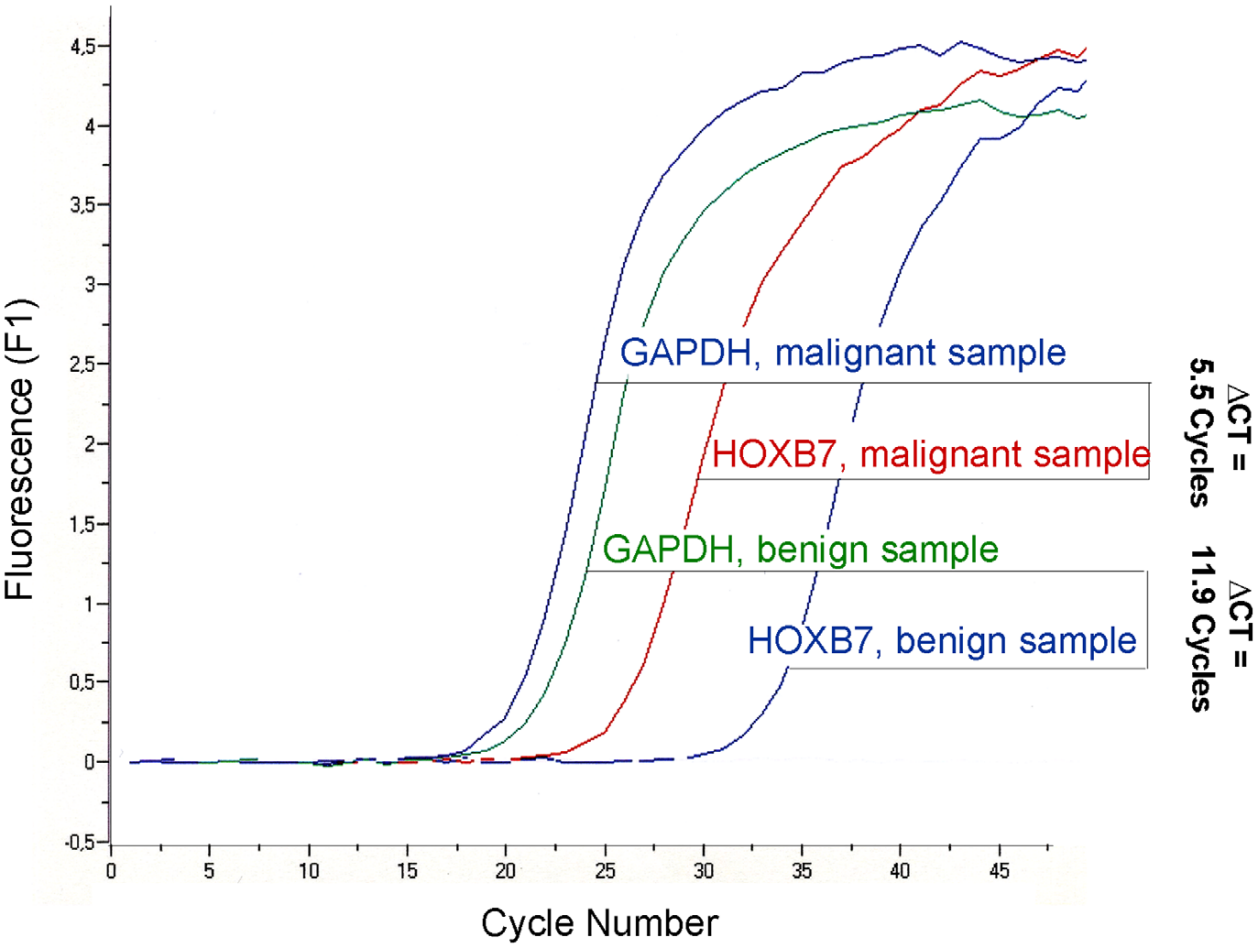
Amplification curve analysis of a representative benign and malignant sample, respectively.

To compare target gene-specific transcripts in malignant samples to those in benign samples, their Ct (threshold cycle) values were normalized in a first step by subtracting the Ct value of the GAPDH control (ΔCt = Ct, target – Ct, control) [40]. For representative samples see figure 1. Over-expression of gene-specific mRNA in malignant samples was calculated by subtracting the normalized median ΔCt of malignant samples and benign samples (ΔΔCt = median ΔCt, malignant – median ΔCt, benign). Overexpression ratios were calculated as 2^ΔΔCt^ [41].

### Statistical Analysis

Statistical analysis was performed with SPSS software version 17.0.0 (SPSS Inc., Chicago) and GraphPad Prism for Windows version 4.00. Data were checked for normal distribution, and compared by two-tailed t-tests as appropriate using JavaStat (http://statpages.org/ctab2x2.html). Data are reported as means ± standard deviations, unless stated otherwise. Correlations were determined by non-parametric Spearman rank correlation. ROC analysis was performed with the MedCalc software (version 7.3.01). Results with P<0.05 were regarded as statistically significant.

## Results

Based on our classification criteria biliary strictures were considered to represent malignant and benign disease in 72 and 47 patients, respectively. Thirty-one of the 72 patients with malignant strictures had been correctly detected by routine cytology alone (43.1% sensitivity) and malignancy had definitely been excluded in 40 of the 47 patients with benign strictures (85.1% specificity). However, routine cytology failed to detect malignancy in 22 patients and remained indeterminate in further 19 patients, so that overall a correct diagnosis of malignancy was missed by cytology in 41 of 72 patients.

### Messenger-RNA Expression of Candidate Genes

**Figure 2:**
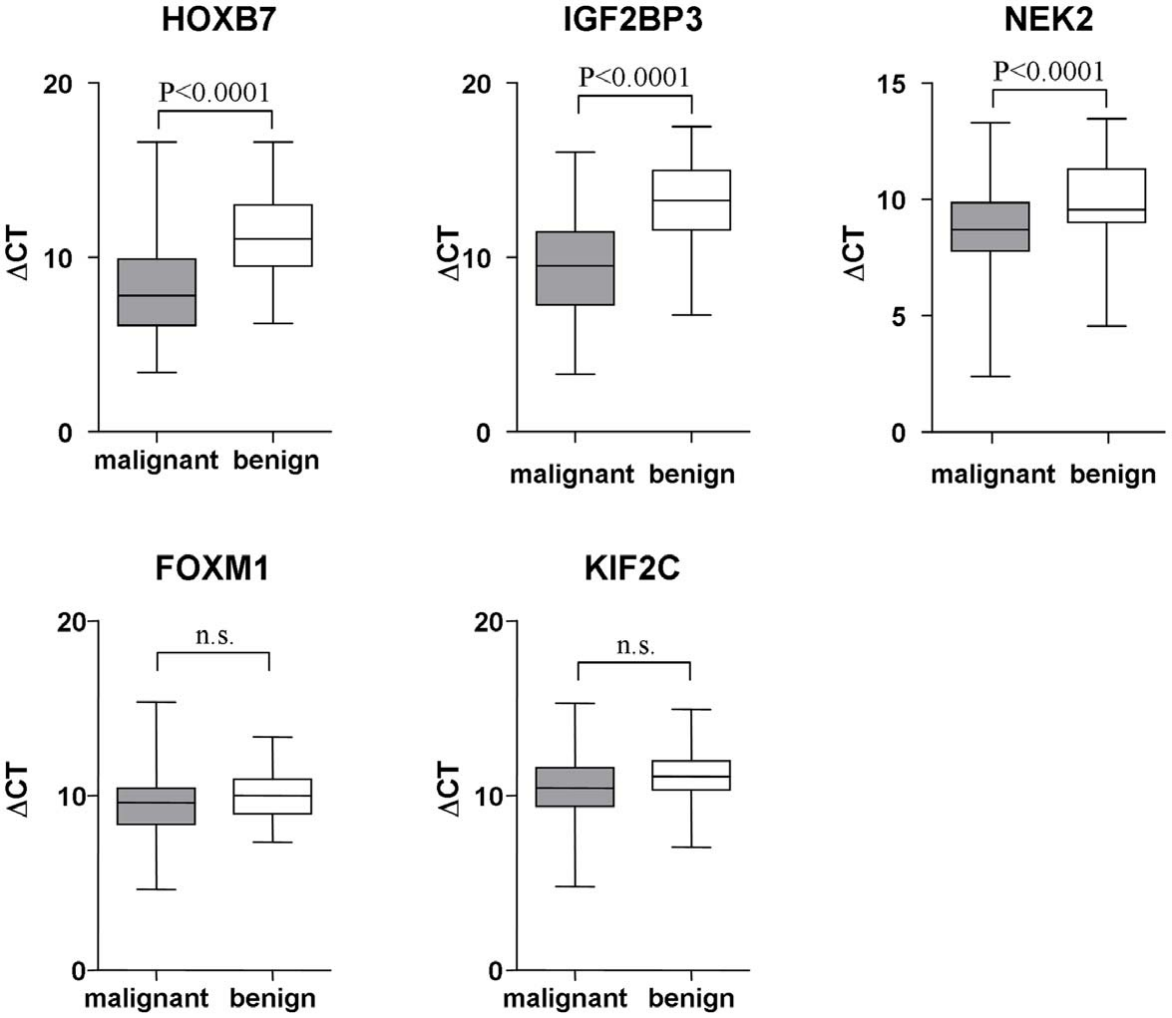
Differences in threshold cycle numbers between target genes (HOXB7, IGF2BP3, NEK2, FOXM1, KIF2C) and the house-keeping gene GAPDH (ΔCT) between patients with malignant (grey boxes) and benign (white boxes) biliary strictures. Results are shown as Box plots with (10-, 25-, 50-, 75-, and 90-percentiles).

Sufficient mRNA could be extracted in each analyzed brush cytology specimen, and target gene mRNA expression levels of IGF2BP3, HOXB7, and NEK2 were significantly higher in the brush specimens from patients with malignant strictures than in those from patients with benign biliary strictures (ΔCt IGF2BP3: 9.58±2.87 vs.13.08±2.38, p<0.0001; ΔCt HOXB7: 8.34±3.08 vs. 11.13±2.51, p<0.0001; ΔCt NEK2: 8.60±1.86 vs. 9.91±1.81, p<0.0001) (figure 2). Median ΔCt values differed by 3.5 cycles (ΔΔCt IGF2BP3), 2.8 cycles (ΔΔCt HOXB7) and 1.3 cycles (ΔΔCt NEK2), indicating approximately 11-fold, 7-fold and 2.5-fold increased mRNA levels in the malignant samples compared to benign strictures. In contrast, mRNA expression levels of FOXM1 and KIF2C did not differ significantly between the groups (ΔCt FOXM1: 9.41±1.83 vs. 10.00±1.23, p<0.13; ΔCt KIF2C: 10.28±2.04 vs. 11.13±1.55, p<0.06) (figure 2). Therefore, these markers were excluded from the further analysis.

In patients with malignant strictures, there was a significant positive correlation between the mRNA expression levels of IGF2BP3, NEK2 and HOXB7 (table 2). In contrast, no correlations between any two markers were found in patients with benign strictures (table 3).

**Table 2:** Correlation between candidate gene ΔCT levels in patients with malignant strictures.

	ΔCT HOXB7	ΔCT NEK2	ΔCT IGF2BP3
**ΔCT HOXB7** Correlation (Spearman)	1	0.259 (*)	0.602 (**)
Significance (2-sided)	−	0.028	0.0001
N	72	72	72
**ΔCT NEK2** Correlation (Spearman)	0.259 (*)	1	0.333 (**)
Significance (2-sided)	0.028	−	0.004
N	72	72	72
**ΔCT IGF2BP3** Correlation (Spearman)	0.602 (**)	0.333 (**)	1
Significance (2-sided)	0.0001	0.004	−
N	72	72	72

(*) Correlation is significant at a niveau of 0.05 (2-sided).

(**) Correlation is significant at a niveau of 0.01 (2-sided).

**Table 3:** Correlation between candidate gene ΔCT levels in patients with benign strictures.

	ΔCT HOXB7	ΔCT NEK2	ΔCT IGF2BP3
**ΔCT HOXB7** Correlation (Spearman)	1	0.158	−0.086
Significance (2-sided)	−	0.290	0.564
N	47	47	47
**ΔCT NEK2** Correlation (Spearman)	0.158	1	0.273
Significance (2-sided)	0.290	−	0.064
N	47	47	47
**ΔCT IGF2BP3** Correlation (Spearman)	−0.086	0.273	1
Significance (2-sided)	0.564	0.064	−
N	47	47	47

(*) Correlation is significant at a niveau of 0.05 (2-sided).

(**) Correlation is significant at a niveau of 0.01 (2-sided).

Finally we checked if baseline differences in gender distribution and bilirubin levels had any effects on gene expression levels. Statistical analysis by independent t-test and Spearman rank correlation did not provide evidence that sex or bilirubin levels were associated with expression levels of IGF2BP3, NEK2 and HOXB7 (data not shown).

### Diagnostic Precision of Single Candidate Genes

**Figure 3:**
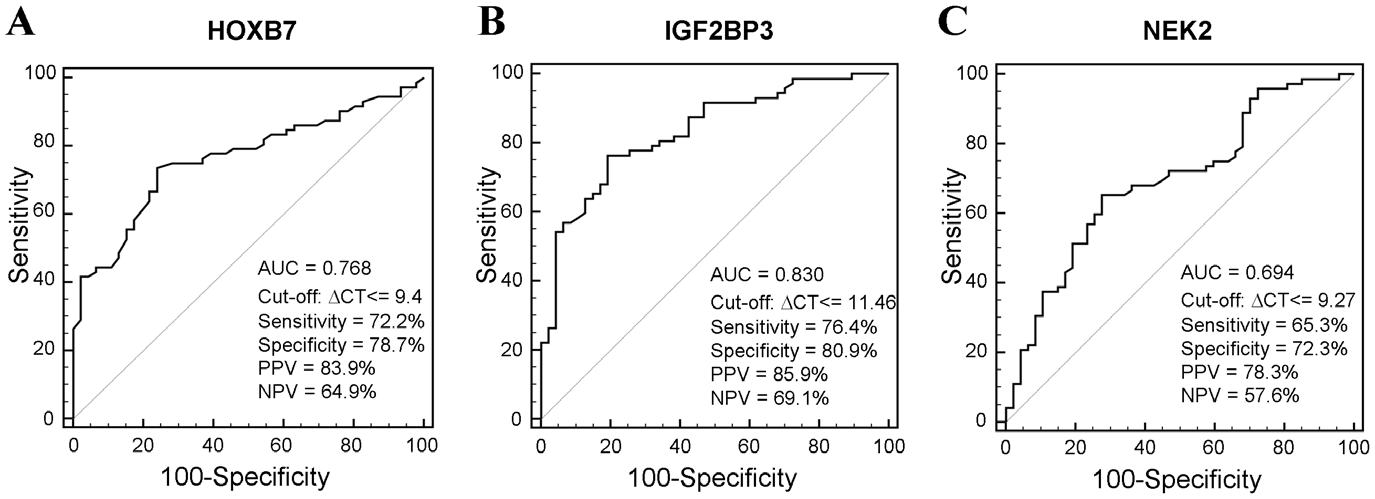
ROC analysis for HOXB7 (figure A), IGF2BP3 (figure B) and NEK2 (figure C) as diagnostic markers for biliary malignancy in patients with biliary strictures. Graphs show sensitivity plotted versus 100% - specificity.

To assess the diagnostic precision of up-regulated expression for individual candidate genes, we next analyzed our data by receiver operating characteristic (ROC) (figure 3). Concerning HOXB7, the area under the ROC curve (AUC) was 0.768 (standard error [SE]: 0.044; 95% CI 0.681–0.841). The optimal cut-off value for HOXB7 was ΔCt < = 9.4, which corresponded to 72.2% [95% CI 65.4–77.5] sensitivity and 78.7% [95% CI 68.3–86.8] specificity. Using the prevalence of our patient cohort, we calculated a positive predictive value (PPV) of 83.9% and a negative predictive value (NPV) of 64.9% (figure 3A). For IGF2BP3 the AUC was 0.830 (SE: 0.038; 95% CI 0.75–0.89), with an optimal cut-off value at ΔCt < = 11.46 (sensitivity: 76.4% [95% CI 69.8–81.4]; specificity: 80.9% [95% CI 70.7–88.5]; PPV: 85.9%; NPV: 69.1%) (figure 3B). ROC analysis of NEK2 yielded an AUC of 0.694 (SE: 0.050; 95% CI 0.602–0.775) with an optimal cut-off value of ΔCt < = 9.27 (sensitivity: 65.3% [95% CI 58.2–71.2]; specificity 72.3% [95% CI 61.5–81.5]; PPV: 78.3%; NPV: 57.6%) (figure 3C).

### Operative Performance of Combined Genetic Markers

To improve diagnostic precision we next analyzed, whether sensitivity and specificity was improved if the results of the single target genes were analyzed in combination. In a first analysis the combination of two tests was considered positive when at least one candidate gene was positive (ΔCt level below the cut-off level) and negative when ΔCt were above the cut-off for both genes. As expected, combined analysis improved sensitivity and decreased specificity for the detection of malignant strictures as compared to the analysis of single parameters:

The combination of IGF2BP3 and NEK2 achieved the best sensitivity (90.3%) but only poor specificity (59.6%). The combination of HOXB7 and NEK2 reached a sensitivity of 87.5% with a specificity of 59.6%, whereas the combination of IGF2BP3 and HOXB7 had slightly lower sensitivity (86.1%) with a specificity of 61.7%.

In an alternative analysis of our data a combination of two test results was considered positive when both candidate genes were positive (ΔCt level below the cut-off level); and negative when at least one or both genes were negative. In this second analysis specificities were increased to 91.5–97.9% but sensitivities dropped to 50.0–62.5%.

Best diagnostic precision was observed, when results of all three target genes were analyzed together and a combination was considered positive if ΔCt levels of any two genes were below the cut-off value (table 4). In this setting 77.8% sensitivity at 87.2% specificity was achieved, which enabled correct benign and malignant diagnoses in 81.5% of all patients. No significant differences could be observed when patients with cholangiocarcinomas (sensitivity 75.0 [66.8–80.5]; specificity 87.2 [78.2–93.3]; accuracy 80.8 [72.2–86.6]) (table 5) and pancreatic cancers (sensitivity 85.0 [68.8–94.1]; specificity 87.2 [80.4–91.1]; accuracy 86.6 [76.9–92.0]) (table 6) were analyzed separately.

**Table 4:** Performance Characteristics for Entire Group (CCC and Pancreatic Cancer) (n = 72).

	Cytology		Single gene expression			Combined gene expression profiles			Cytology/Gene expression#
	M = M	S&M = M	HOXB7	IGF2BP3	NEK2	1 positive	2 positive	3 positive	
Sensitivity (%)	16.7	43.1	72.2	76.4	65.3	93.1	77.8	43.1	87.5
(95% CI)	(11.3–21.1)	(36.5–47.7)	(65.4–77.5)	(69.8–81.4)	(58.2–71.2)	(87.2–96.8)	(71.5–81.9)	(38.1–44.2)	(81.7–91.3)
Specificity (%)	85.1	85.1	78.7	80.9	72.3	46.8	87.2	97.9	87.2
(95% CI)	(76.9–92.0)	(75.1–92.2)	(68.3–86.8)	(70.7–88.5)	(61.5–81.5)	(37.8–52.5)	(77.7–93.5)	(90.3–99.6)	(88.3–93.1)
PPV (%)	63.2	81.6	83.9	85.9	78.3	72.8	90.3	96.9	91.3
(95% CI)	(42.9–80.1)	(69.2–90.3)	(76.0–90.0)	(78.5–91.5)	(69.8–85.5)	(68.2–75.79)	(83.1–95.1)	(85.7–99.4)	(85.2–95.3)
NPV (%)	40.0	49.4	64.9	69.1	57.6	81.5	71.9	52.9	82.0
(95% CI)	(36.1–43.2)	(43.6–53.5)	(56.3–71.6)	(60.4–75.6)	(49.0–64.9)	(65.8–91.4)	(64.1–77.1)	(48.8–53.8)	(73.6–87.3)
Accuracy (%)	43.7	59.7	74.8	78.2	68.1	74.8	81.5	64.7	87.4
(95% CI)	(37.2–49.1)	(51.8–65.2)	(66.5–81.2)	(70.14–84.2)	(59.5–75.3)	(67.7–79.3)	(74.0–86.5)	(58.7–66.1)	(80.3–92.0)

M = malignant; S&M = suspicious and malignant.

# Cytology: M = S&M; Gene expression: 2 of 3 positive.

**Table 5:** Performance Characteristics for Patients with CCC (n = 52).

	Cytology		Single gene expression			Combined gene expression profiles			Cytology/Gene expression#
	M = M	S&M = M	HOXB7	IGF2BP3	NEK2	1 positive	2 positive	3 positive	
Sensitivity (%)	21.2	53.8	71.2	76.9	71.2	94.2	75.0	50.0	88.5
(95% CI)	(14.0–27.3)	(45.3–60.1)	(62.3–78.1)	(68.3–83.4)	(62.1–78.7)	(86.8–97.9)	(66.8–80.5)	(43.4–51.6)	(81.0–93.3)
Specificity (%)	85.1	85.1	78.7	80.9	72.3	46.8	87.2	97.9	87.2
(95% CI)	(77.2–91.9)	(75.6–92.0)	(68.9–86.4)	(71.4–88.0)	(62.3–80.7)	(38.6–50.9)	(78.2–93.3)	(90.6–99.6)	(78.9–92.6)
PPV (%)	61.1	80.0	78.7	81.6	74.0	66.2	86.7	96.3	88.5
(95% CI)	(40.5–78.7)	(87.3–89.2)	(68.9–86.4)	(72.5–88.5)	(64.6–81.8)	(61.0–68.8)	(77.2–93.1)	(83.6–99.3)	(81.0–93.3)
NPV (%)	49.4	62.5	71.2	76.0	69.4	88.0	75.9	63.9	87.2
(95% CI)	(44.8–53.3)	(55.5–67.5)	(62.3–78.1)	(67.1–82.7)	(59.8–77.4)	(72.6–95.7)	(68.0–81.2)	(59.1–65.0)	(78.9–92.6)
Accuracy (%)	51.5	68.7	74.7	78.8	71.7	71.7	80.8	72.7	87.9
(95% CI)	(44.0–57.9)	(59.7–75.2)	(65.4–82.1)	(69.8–85.6)	(62.2–79.6)	(63.9–75.6)	(72.2–86.6)	(65.8–74.4)	(80.0–92.9)

M = malignant; S&M = suspicious and malignant.

# Cytology: M = S&M; Gene expression: 2 of 3 positive.

**Table 6:** Performance Characteristics for Patients with Pancreatic Cancer (n = 20).

	Cytology		Single gene expression			Combined gene expression profiles			Cytology/Gene expression#
	M = M	S&M = M	HOXB7	IGF2BP3	NEK2	1 positive	2 positive	3 positive	
Sensitivity (%)	5.0	15.0	75.0	75.0	50.0	90.0	85.0	25.0	85.0
(95% CI)	(0.9–17.8)	(5.6–28.8)	(57.3–87.5)	(57.5–87.3)	(32.7–66.5)	(73.3–97.1)	(68.8–94.1)	(13.7–29.1)	(68.8–94.1)
Specificity (%)	85.1	85.1	78.7	80.9	72.3	46.8	87.2	97.9	87.2
(95% CI)	(83.4–90.5)	(81.1–91.0)	(71.2–84.0)	(73.4–86.1)	(65.0–79.3)	(39.7–49.8)	(80.4–91.1)	(93.1–99.6)	(80.4–91.1)
PPV (%)	12.5	30.0	60.0	62.5	43.5	41.9	73.9	83.3	73.9
(95% CI)	(2.3–44.4)	(11.2–57.6)	(45.9–70.0)	(47.9–72.8)	(28.4–57.8)	(34.1–45.2)	(59.9–81.8)	(45.7–97.0)	(59.8–81.8)
NPV (%)	67.8	70.2	88.1	88.4	77.3	91.7	93.2	75.4	93.2
(95% CI)	(66.4–72.1)	(66.9–75.0)	(79.7–94.0)	(80.2–94.1)	(69.4–84.8)	(77.8–97.6)	(85.8–97.3)	(71.7–76.8)	(85.8–97.3)
Accuracy (%)	61.2	64.2	77.6	79.1	65.7	59.7	86.6	76.1	86.6
(95% CI)	(58.7–72.4)	(58.6–72.4)	(67.1–85.1)	(68.7–86.5)	(55.3–75.5)	(49.7–64.0)	(76.9–92.0)	(69.4–78.6)	(76.9–92.0)

M = malignant; S&M = suspicious and malignant.

# Cytology: M =  S&M; Gene expression: 2 of 3 positive.

Diagnostic precision was even more increased, when cytology results were analyzed together with up-regulated expression of our three target genes. Brush cytology results together with detection of increased HOXB7, IGF2BP3 and NEK2 mRNA reached 87.5% sensitivity (63 of 72) and 87.2% specificity (41 of 47), corresponding to a PPV of 91.3% and a NPV of 82.0%. Finally a diagnostic accuracy of 87.4% was achieved which enabled a correct diagnosis in 104 of the 119 patients.

## Discussion

Correct diagnosis of bile duct strictures is a challenge, taking into account that current techniques with intraductal brush cytology and biopsies have low diagnostic yields [3], [6], [7], [8], [9].

Therefore, new diagnostic approaches are needed to reliably distinguish malignant from benign biliary strictures to offer patients appropriate therapy. Measuring tumor-associated gene expression in biliary tract specimens is a potentially useful tool for a reliable diagnosis of bile duct cancer.

Taking advantage of two recent studies which had investigated global gene expression patterns in human cholangiocarcinomas, we selected IGF2BP3, NEK2, and HOXB7 as candidate target genes and studied their mRNA expression levels in brush cytology specimens because robust up-regulation of these genes had consistently been found in biliary cancer [16], [17].

In line with previous studies, we found significantly higher mRNA expression levels of IGF2BP3, HOXB7, and NEK2 in brush cytology specimens from patients with malignant disease than in those obtained from patients with benign biliary strictures which enabled us to define cut-off values by ROC analysis.

Based on these cut-off values, we next stratified gene expression levels in positive (ΔCt below cut-off) and negative (ΔCt above the cut-off) and calculated sensitivities and specificities to detect bile duct malignancy. By this approach sensitivities of single genetic marker genes ranged from 65.3–76.4% with specificities between 72.3–80.9%. Routine brush cytology also achieved a high specificity (85.1%). However, sensitivity of cytological diagnostic was low (16.7–43.7%). This was in good agreement with previously published data concerning diagnostic accuracy of cytology [9], since most other authors also had reported good specificity but insufficient sensitivity [8], [9], [42]. Of note, analysis of expression levels of marker genes also enabled identification of pancreatic cancers as underlying disease of biliary strictures with high accuracy whereas diagnostic performance of routine brush cytology was insufficient (sensitivity 5–15%). Moreover, cytological interpretation is also hampered by prior biliary manipulations such as stenting which can affect the morphology of cells in cytology smears [43], whereas such procedures do not seem to affect expression levels of the selected target genes.

Both study groups (patients with malignant and benign strictures) differed significantly with respect to gender distribution and bilirubin levels. However, there was no association between target gene expression levels and patient gender or serum bilirubin. Thus, it is unlikely that differences in the composition of both study populations biased our results.

Up-regulated expression of IGF2BP3, NEK2 and HOXB7 mRNA in our study confirms recent reports suggesting detection of tumor-associated gene expression profiles in biliary tract specimens as potentially useful tools in the diagnosis of bile duct cancer. For example, telomerase mRNA was detected by in situ hybridization in 6/8 brushing samples from patients with bile duct cancer and a correct diagnosis was possible in all patients after combination with results of routine cytology [44]. Other authors have reported specific detection of telomerase activity in 11/13 (85%) and 6/8 (75%) biopsy samples from patients with bile duct carcinoma [45], [46]. Likewise, analysis of peptide profiles in bile by capillary electrophoresis/mass spectrometry has recently been validated to reliably differentiate between benign and malignant biliary lesions [47]. However, these novel detection techniques are rather time and cost intensive and are available at only few locations. Alternatively, Chapman and colleagues recently demonstrated that - despite rather poor integrity - mRNA isolated from brushings of macroscopically normal bile ducts or benign strictures (n = 4) and malignant biliary strictures (n = 6) is nevertheless suited for molecular analysis of biliary pathology using sensitive qPCR and microarray techniques [48]. Differential gene expression by microarray analysis identified 1140 up-regulated genes and 1001 down-regulated genes between benign and malignant biliary strictures. In a selection of 45 up-regulated genes including HOX genes microarray results were validated by qPCR. Our study extends this strategy to a considerably greater number of patients and confirms that combining molecular analysis of selected genetic markers with conventional cytology can markedly improve diagnostic identification of patients with malignant biliary disorders.

In contrast, immunostaining for p53 overexpression in biliary brush cytology specimens did not increase the diagnostic accuracy for bile duct cancer in two separate studies with 10 and 13 patients, respectively [42], [49]. A variety of other markers have been evaluated in order to improve the diagnosis of bile duct carcinoma in bile samples but in general sensitivity and specificity have remained disappointing (reviewed in [50]). Shi et al. recently analyzed KRAS2 mutations in neoplastic and non-neoplastic pancreatobiliary diseases [51]. In this study KRAS2 mutations were detected in 14/16 (87.5%) malignant samples and 9/28 (32.1%) benign samples, with significantly higher mutation levels in the neo-plastic samples. However, in a subsequent study Kipp et al. showed that KRAS mutations could be found in only 29% of cholangiocarcinoma specimens as compared to 69% of pancreatic adenocarcinoma samples [52]. Thus, KRAS mutation analysis might be of limited diagnostic value in patients with biliary strictures caused by non-pancreatic malignancies. In our study, however, diagnostic performance of target gene expression analysis was similar in patients with cholangicarcinomas and pancreatic cancers.

Diagnostic sensitivity of routine cytology was also increased by using fluorescence in-situ hybridization (FISH) and digital image analysis (DIA) on specimens from fine needle aspirations [53]. When applied to brush cytology FISH may also increase cancer detection rate in patients with biliary strictures [54]. However, in the study by Kipp et al. polysomic FISH results could be identified in only 41% (17/41) of cholangiocarcinoma specimens [52].

On the other hand, LightCycler-based RT-PCR determination of HOXB7, IGF2BP3 and NEK2 mRNA provides a quick, inexpensive, and reliable method, because all genes can be processed simultaneously in the same amplification protocol. For instance, in our study it took only 2 hours for entire sample processing and approximately 20 € per patient to obtain the results from the molecular analysis. Apart from improved diagnostic performance the novel real time RT-PCR method has further advantages. Compared to intraductal biopsies and fine needle aspirations, brush cytology does not require additional effort for sample acquisition and thus carries a low complication risk. It can be applied also to tight strictures with relative ease and probably reflects a low sampling error [8]. However, future prospective studies in independent cohorts are warranted to confirm our results.

In conclusion, our data suggest that RT-PCR based detection of IGF2BP3, NEK2, and HOXB7-mRNA from intraductal brush cytology specimens may become a valuable additional tool that in combination with routine cytological examination has promising potential to improve the overall diagnostic yield of intraductal brush cytology in patients with bile duct strictures.

## References

[1] Menias CO, Surabhi VR, Prasad SR, Wang HL, Narra VR, et al (2008) Mimics of cholangiocarcinoma: spectrum of disease. Radiographics 28: 1115–1129. 18635632 10.1148/rg.284075148

[2] Hann LE, Winston CB, Brown KT, Akhurst T (2000) Diagnostic imaging approaches and relationship to hepatobiliary cancer staging and therapy. Semin Surg Oncol 19: 94–115. 11126385 10.1002/1098-2388(200009)19:2<94::aid-ssu3>3.0.co;2-x

[3] Pugliese V, Conio M, Nicolo G, Saccomanno S, Gatteschi B (1995) Endoscopic retrograde forceps biopsy and brush cytology of biliary strictures: a prospective study. Gastrointest Endosc 42: 520–526. 8674921 10.1016/s0016-5107(95)70004-8

[4] Schoefl R, Haefner M, Wrba F, Pfeffel F, Stain C, et al (1997) Forceps biopsy and brush cytology during endoscopic retrograde cholangiopancreatography for the diagnosis of biliary stenoses. Scand J Gastroenterol 32: 363–368. 9140159 10.3109/00365529709007685

[5] Glasbrenner B, Ardan M, Boeck W, Preclik G, Moller P, et al (1999) Prospective evaluation of brush cytology of biliary strictures during endoscopic retrograde cholangiopancreatography. Endoscopy 31: 712–717. 10604612 10.1055/s-1999-73

[6] Ryan ME, Baldauf MC (1994) Comparison of flow cytometry for DNA content and brush cytology for detection of malignancy in pancreaticobiliary strictures. Gastrointest Endosc 40: 133–139. 8013809 10.1016/s0016-5107(94)70154-7

[7] Ryan ME (1991) Cytologic brushings of ductal lesions during ERCP. Gastrointest Endosc 37: 139–142. 1851708 10.1016/s0016-5107(91)70671-8

[8] de Bellis M, Sherman S, Fogel EL, Cramer H, Chappo J, et al (2002) Tissue sampling at ERCP in suspected malignant biliary strictures (Part 2). Gastrointest Endosc 56: 720–730. 12397282 10.1067/mge.2002.129219

[9] De Bellis M, Sherman S, Fogel EL, Cramer H, Chappo J, et al (2002) Tissue sampling at ERCP in suspected malignant biliary strictures (Part 1). Gastrointest Endosc 56: 552–561. 12297773 10.1067/mge.2002.128132

[10] Principe A, Ercolani G, Bassi F, Paolucci U, Raspadori A, et al (2003) Diagnostic dilemmas in biliary strictures mimicking cholangiocarcinoma. Hepatogastroenterology 50: 1246–1249. 14571710

[11] Fishman DS, Tarnasky PR, Patel SN, Raijman I (2009) Management of pancreaticobiliary disease using a new intra-ductal endoscope: the Texas experience. World J Gastroenterol 15: 1353–1358. 19294765 10.3748/wjg.15.1353 PMC2658829

[12] Siddiqui AA, Mehendiratta V, Jackson W, Loren DE, Kowalski TE, et al (2012) Identification of cholangiocarcinoma by using the Spyglass Spyscope system for peroral cholangioscopy and biopsy collection. Clin Gastroenterol Hepatol 10: 466–471; quiz e448. 22178463 10.1016/j.cgh.2011.12.021

[13] Takano T, Miyauchi A, Matsuzuka F, Liu G, Higashiyama T, et al (1999) Preoperative diagnosis of medullary thyroid carcinoma by RT-PCR using RNA extracted from leftover cells within a needle used for fine needle aspiration biopsy. J Clin Endocrinol Metab 84: 951–955. 10084577 10.1210/jcem.84.3.5558

[14] Takano T, Miyauchi A, Yokozawa T, Matsuzuka F, Maeda I, et al (1999) Preoperative diagnosis of thyroid papillary and anaplastic carcinomas by real-time quantitative reverse transcription-polymerase chain reaction of oncofetal fibronectin messenger RNA. Cancer Res 59: 4542–4545. 10493503

[15] Feldmann G, Nattermann J, Nischalke HD, Gorschluter M, Kuntzen T, et al (2006) Detection of human aspartyl (asparaginyl) beta-hydroxylase and homeobox B7 mRNA in brush cytology specimens from patients with bile duct cancer. Endoscopy 38: 604–609. 16673309 10.1055/s-2006-925065

[16] Obama K, Ura K, Li M, Katagiri T, Tsunoda T, et al (2005) Genome-wide analysis of gene expression in human intrahepatic cholangiocarcinoma. Hepatology 41: 1339–1348. 15880566 10.1002/hep.20718

[17] Kokuryo T, Senga T, Yokoyama Y, Nagino M, Nimura Y, et al (2007) Nek2 as an effective target for inhibition of tumorigenic growth and peritoneal dissemination of cholangiocarcinoma. Cancer Res 67: 9637–9642. 17942892 10.1158/0008-5472.CAN-07-1489

[18] Fraser MM, Watson PM, Fraig MM, Kelley JR, Nelson PS, et al (2005) CaSm-mediated cellular transformation is associated with altered gene expression and messenger RNA stability. Cancer Res 65: 6228–6236. 16024624 10.1158/0008-5472.CAN-05-0650

[19] Katoh Y, Katoh M (2009) Hedgehog target genes: mechanisms of carcinogenesis induced by aberrant hedgehog signaling activation. Curr Mol Med 9: 873–886. 19860666 10.2174/156652409789105570

[20] Agnelli L, Storti P, Todoerti K, Sammarelli G, Dalla Palma B, et al (2011) Overexpression of HOXB7 and homeobox genes characterizes multiple myeloma patients lacking the major primary immunoglobulin heavy chain locus translocations. Am J Hematol 86: E64–66. 21953534 10.1002/ajh.22164

[21] Suzuki K, Kokuryo T, Senga T, Yokoyama Y, Nagino M, et al (2010) Novel combination treatment for colorectal cancer using Nek2 siRNA and cisplatin. Cancer Sci 101: 1163–1169. 20345485 10.1111/j.1349-7006.2010.01504.x PMC11159639

[22] Wu X, Chen H, Parker B, Rubin E, Zhu T, et al (2006) HOXB7, a homeodomain protein, is overexpressed in breast cancer and confers epithelial-mesenchymal transition. Cancer Res 66: 9527–9534. 17018609 10.1158/0008-5472.CAN-05-4470

[23] Kobel M, Xu H, Bourne PA, Spaulding BO, Shih Ie M, et al (2009) IGF2BP3 (IMP3) expression is a marker of unfavorable prognosis in ovarian carcinoma of clear cell subtype. Mod Pathol 22: 469–475. 19136932 10.1038/modpathol.2008.206

[24] Wang S, Li W, Lv S, Wang Y, Liu Z, et al (2011) Abnormal expression of Nek2 and beta-catenin in breast carcinoma: clinicopathological correlations. Histopathology 59: 631–642. 22014044 10.1111/j.1365-2559.2011.03941.x

[25] Gnjatic S, Cao Y, Reichelt U, Yekebas EF, Nolker C, et al (2010) NY-CO-58/KIF2C is overexpressed in a variety of solid tumors and induces frequent T cell responses in patients with colorectal cancer. Int J Cancer 127: 381–393. 19937794 10.1002/ijc.25058

[26] Chan DW, Yu SY, Chiu PM, Yao KM, Liu VW, et al (2008) Over-expression of FOXM1 transcription factor is associated with cervical cancer progression and pathogenesis. J Pathol 215: 245–252. 18464245 10.1002/path.2355

[27] Gemenetzidis E, Bose A, Riaz AM, Chaplin T, Young BD, et al (2009) FOXM1 upregulation is an early event in human squamous cell carcinoma and it is enhanced by nicotine during malignant transformation. PLoS One 4: e4849. 19287496 10.1371/journal.pone.0004849 PMC2654098

[28] Ligato S, Zhao H, Mandich D, Cartun RW (2008) KOC (K homology domain containing protein overexpressed in cancer) and S100A4-protein immunoreactivity improves the diagnostic sensitivity of biliary brushing cytology for diagnosing pancreaticobiliary malignancies. Diagn Cytopathol 36: 561–567. 18618724 10.1002/dc.20836

[29] Zhao H, Mandich D, Cartun RW, Ligato S (2007) Expression of K homology domain containing protein overexpressed in cancer in pancreatic FNA for diagnosing adenocarcinoma of pancreas. Diagn Cytopathol 35: 700–704. 17924416 10.1002/dc.20739

[30] Rodriguez JA, Li M, Yao Q, Chen C, Fisher WE (2005) Gene overexpression in pancreatic adenocarcinoma: diagnostic and therapeutic implications. World J Surg 29: 297–305. 15696394 10.1007/s00268-004-7843-0

[31] Schaeffer DF, Owen DR, Lim HJ, Buczkowski AK, Chung SW, et al (2010) Insulin-like growth factor 2 mRNA binding protein 3 (IGF2BP3) overexpression in pancreatic ductal adenocarcinoma correlates with poor survival. BMC Cancer 10: 59. 20178612 10.1186/1471-2407-10-59 PMC2837867

[32] Wang Z, Ahmad A, Banerjee S, Azmi A, Kong D, et al (2010) FoxM1 is a novel target of a natural agent in pancreatic cancer. Pharm Res 27: 1159–1168. 20354770 10.1007/s11095-010-0106-x PMC2975383

[33] Gray S, Pandha HS, Michael A, Middleton G, Morgan R (2011) HOX genes in pancreatic development and cancer. Jop 12: 216–219. 21546695

[34] Wonsey DR, Follettie MT (2005) Loss of the forkhead transcription factor FoxM1 causes centrosome amplification and mitotic catastrophe. Cancer Res 65: 5181–5189. 15958562 10.1158/0008-5472.CAN-04-4059

[35] Wang Z, Banerjee S, Kong D, Li Y, Sarkar FH (2007) Down-regulation of Forkhead Box M1 transcription factor leads to the inhibition of invasion and angiogenesis of pancreatic cancer cells. Cancer Res 67: 8293–8300. 17804744 10.1158/0008-5472.CAN-07-1265

[36] Yantiss RK, Woda BA, Fanger GR, Kalos M, Whalen GF, et al (2005) KOC (K homology domain containing protein overexpressed in cancer): a novel molecular marker that distinguishes between benign and malignant lesions of the pancreas. Am J Surg Pathol 29: 188–195. 15644775 10.1097/01.pas.0000149688.98333.54

[37] Shimo A, Tanikawa C, Nishidate T, Lin ML, Matsuda K, et al (2008) Involvement of kinesin family member 2C/mitotic centromere-associated kinesin overexpression in mammary carcinogenesis. Cancer Sci 99: 62–70. 17944972 10.1111/j.1349-7006.2007.00635.x PMC11158784

[38] Nakamura Y, Tanaka F, Haraguchi N, Mimori K, Matsumoto T, et al (2007) Clinicopathological and biological significance of mitotic centromere-associated kinesin overexpression in human gastric cancer. Br J Cancer 97: 543–549. 17653072 10.1038/sj.bjc.6603905 PMC2360338

[39] Hansel DE, Rahman A, Hidalgo M, Thuluvath PJ, Lillemoe KD, et al (2003) Identification of novel cellular targets in biliary tract cancers using global gene expression technology. Am J Pathol 163: 217–229. 12819026 10.1016/S0002-9440(10)63645-0 PMC1868162

[40] Mori R, Wang Q, Danenberg KD, Pinski JK, Danenberg PV (2008) Both beta-actin and GAPDH are useful reference genes for normalization of quantitative RT-PCR in human FFPE tissue samples of prostate cancer. Prostate 68: 1555–1560. 18651557 10.1002/pros.20815

[41] Weglarz L, Molin I, Orchel A, Parfiniewicz B, Dzierzewicz Z (2006) Quantitative analysis of the level of p53 and p21(WAF1) mRNA in human colon cancer HT-29 cells treated with inositol hexaphosphate. Acta Biochim Pol 53: 349–356. 16733561

[42] Ponsioen CY, Vrouenraets SM, van Milligen de Wit AW, Sturm P, Tascilar M, et al (1999) Value of brush cytology for dominant strictures in primary sclerosing cholangitis. Endoscopy 31: 305–309. 10376457 10.1055/s-1999-18

[43] Rupp M, Hawthorne CM, Ehya H (1990) Brushing cytology in biliary tract obstruction. Acta Cytol 34: 221–226. 2321454

[44] Morales CP, Burdick JS, Saboorian MH, Wright WE, Shay JW (1998) In situ hybridization for telomerase RNA in routine cytologic brushings for the diagnosis of pancreaticobiliary malignancies. Gastrointest Endosc 48: 402–405. 9786114 10.1016/s0016-5107(98)70011-2

[45] Itoi T, Shinohara Y, Takeda K, Takei K, Ohno H, et al (2000) Detection of telomerase activity in biopsy specimens for diagnosis of biliary tract cancers. Gastrointest Endosc 52: 380–386. 10968854 10.1067/mge.2000.108303

[46] Niiyama H, Mizumoto K, Kusumoto M, Ogawa T, Suehara N, et al (1999) Activation of telomerase and its diagnostic application in biopsy specimens from biliary tract neoplasms. Cancer 85: 2138–2143. 10326691 10.1002/(sici)1097-0142(19990515)85:10<2138::aid-cncr7>3.0.co;2-8

[47] Lankisch TO, Metzger J, Negm AA, Vosskuhl K, Schiffer E, et al (2011) Bile proteomic profiles differentiate cholangiocarcinoma from primary sclerosing cholangitis and choledocholithiasis. Hepatology 53: 875–884. 21374660 10.1002/hep.24103

[48] Chapman MH, Tidswell R, Dooley JS, Sandanayake NS, Cerec V, et al (2012) Whole genome RNA expression profiling of endoscopic biliary brushings provides data suitable for biomarker discovery in cholangiocarcinoma. J Hepatol. 10.1016/j.jhep.2011.10.022 PMC3307884 22173169

[49] Stewart CJ, Burke GM (2000) Value of p53 immunostaining in pancreatico-biliary brush cytology specimens. Diagn Cytopathol 23: 308–313. 11074623 10.1002/1097-0339(200011)23:5<308::aid-dc4>3.0.co;2-h

[50] Nehls O, Gregor M, Klump B (2004) Serum and bile markers for cholangiocarcinoma. Semin Liver Dis 24: 139–154. 15192787 10.1055/s-2004-828891

[51] Shi C, Chandrasekaran A, Thuluvath PJ, Karikari C, Argani P, et al (2009) Ultrasensitive detection of KRAS2 mutations in bile and serum from patients with biliary tract carcinoma using LigAmp technology. J Mol Diagn 11: 583–589. 19815696 10.2353/jmoldx.2009.090061 PMC2765758

[52] Kipp BR, Barr Fritcher EG, Clayton AC, Gores GJ, Roberts LR, et al (2010) Comparison of KRAS Mutation Analysis and FISH for Detecting Pancreatobiliary Tract Cancer in Cytology Specimens Collected During Endoscopic Retrograde Cholangiopancreatography. J Mol Diagn [Epub ahead of print]. 10.2353/jmoldx.2010.100016 PMC2963905 20864634

[53] Levy MJ, Clain JE, Clayton A, Halling KC, Kipp BR, et al (2007) Preliminary experience comparing routine cytology results with the composite results of digital image analysis and fluorescence in situ hybridization in patients undergoing EUS-guided FNA. Gastrointest Endosc 66: 483–490. 17725938 10.1016/j.gie.2007.03.1053

[54] Fritcher EG, Halling KC (2010) Advanced cytologic approaches for the diagnosis of pancreatobiliary cancer. Curr Opin Gastroenterol 26: 259–264. 20393279 10.1097/MOG.0b013e3283383bd0

